# Prevalence and severity of ocular involvement in Graves’ disease according to sex and age: A clinical study from Babol, Iran

**DOI:** 10.22088/cjim.9.2.178

**Published:** 2018

**Authors:** Sara Gharib, Zoleika Moazezi, Mohammad Ali Bayani

**Affiliations:** 1 Clinical Research Development Unit of Ayatollah Rouhani Hospital, Babol University of Medical Sciences, Babol, Iran.; 2 Cancer Research Center, Health Research Institute, Babol University of Medical Sciences, Babol, Iran.; 3 Social Determinants of Health Research Center, Health Research Institute, Babol University of Medical Sciences, Babol, Iran.

**Keywords:** Graves’ disease, Thyroid-associated eye disease, Proptosis, NO SPECS

## Abstract

**Background:**

Thyroid-associated eye disease (TED), previously known as Graves’ ophthalmopathy is a cosmetically and functionally debilitating disease that is seen worldwide. The aim of this study was to evaluate the prevalence and clinical severity of ocular manifestations of Graves’ disease according to sex, age and duration in northern Iran.

**Methods:**

Between April 2011 and March 2012, 105 patients with Graves’ disease, underwent ophthalmic examination, including ocular motility, exophthalmometry, intraocular pressure (IOP), slit lamp and fundoscopy. Patients received scores according to modified Werner’s NO SPECS classification.

**Results:**

Ocular involvement was found in 70 patients with established Graves’s disease. The mean age was 35.0 years, (SD 13.0, range 15 to 69). The most common ocular findings were exophthalmometric proptosis of more than 20 mm (63.8%), lid lag (55.7%), lid retraction (52.8%) and tearing (38.6%). Almost 70% of patients had bilateral involvement. Elevated IOP was seen in 15 (25.4%) patients, and was significantly related to proptosis (P=0.007). More than half of the patients (n=36, 52.2%) had a modified Werner’s NO SPECS score of 3.00. Clinical severity as shown by the increasing number of signs and symptoms per patient was correlated to increasing age (r=0.31, P=0.01) but not to gender (P=0.17).

**Conclusions:**

Both functional (ocular motility disorders, increased IOP) and cosmetic (proptosis, periorbital edema) sequels are common ocular presentations in patients with Graves' disease. Proptosis was the most common finding in this study and was associated with elevated IOP. Clinical severity was found to correlate to increasing age.

Thyroid-associated eye disease (TED), is a cosmetically disfiguring and functionally incapacitating, chronic, infiltrative ocular disease that is usually associated with Graves' disease (1). However, it may be seen with other thyroid diseases such as Hashimoto’s thyroiditis and thyroid cancer (2). More than 50% of patients with Graves' disease will develop mild to severe, unilateral or bilateral TED at some point during the course of their disease with severe forms affecting 3% to 5% of patients. Onset of eye disease is usually concomitant with the onset of hyperthyroidism, but ophthalmopathy may precede or follow hyperthyroidism and even occur in thyroid or hypothyroid patients (2). TED is seen six times more frequently in females than males (86% versus 14% of cases, respectively), but the female: male ratio is reduced to 4:1 with severe forms of eye disease (3). The Iranian scientist Sayyid Ismail Al-Jurjani first described the relationship between exophthalmos and thyroid disease in the 12th century (2, 4). 

There have been only a few studies on clinical features of TED in Iranian patients (5). In order to determine the prevalence and severity of ophthalmopathy in Graves' patients in the city of Babol in northern Iran, a large number of patients with TED were studied, and the influence of various factors, such as age, sex and thyroid function, on the severity of ophthalmopathy was evaluated. In addition, the relative frequencies of our findings were compared and contrasted with those of previously reported studies. 

## Methods

This was a descriptive cross-sectional study. The study population included all consecutive patients with confirmed diagnosis of Graves' disease who referred to the endocrine clinic of Ayatollah Rouhani Hospital in Babol, Iran, from April 2011 through March 2012. The diagnosis of Graves' disease was based on clinical and laboratory findings of diffuse enlargement of thyroid gland, raised free thyroxin or triiodothyronine levels, and suppressed thyroid-stimulating hormone levels. Information regarding ocular symptoms, family history, smoking history, associated systemic disease and the treatment regimens were obtained. The study had no exclusion criteria and all patients with documented history of Graves' disease with any age, onset or type of intervention were included. A single ophthalmologist performed ocular examinations in all patients.

A comprehensive ophthalmic examination including visual acuity, slit-lamp examination, evaluation of eyelids and ocular movements, tonometry with Keeler non-contact tonometer, exophthalmometry with Hertel exophthalmometer, and fundoscopy for evaluation of optic nerve head was performed for all patients. Best-corrected visual acuity (BCVA) was documented using Snellen chart. Intraocular pressure (IOP) was measured in primary position and upward gaze. Retraction of either upper or lower eyelid was defined as any exposed superior or inferior sclera beyond the limbus in the primary gaze. Proptosis was defined as the measurement of antero-posterior protrusion of the globe 20 mm or more from the orbital rim in either eye or any discrepancy in the degree of protrusion of the two eyes by >2 mm (6). Corneal involvement was assessed with fluorescein staining under slit lamp microscopy. The relation of thyroid size and function with ocular manifestations were also evaluated. The classification of TED was based on modified Werner’s classification, as endorsed by the American Thyroid Association (7) (Table 1). Results were analyzed using the SPSS Version 18 (chi squared, Mann Whitney u, Spearman, Kruskal-Wallis tests).

**Table 1:** Modified Werner’s NO SPECS classification of eye changes in Graves’ ophthalmopathy

**Class**	**Description**
0	No physical signs or symptoms
1	Only signs, no symptoms (upper lid retraction, stare, and eyelid lag)
2	Soft tissue involvement (symptoms and signs)
3	Proptosis
4	Extraocular muscle involvement
5	Corneal involvement
6	Sight Loss (optic nerve involvement)

## Results

A total of 105 patients with established Graves' disease were studied. 70 patients, 26 (37.1%) males and 44 (62.9 %) females, were diagnosed with Graves’ ophthalmopathy (GO). The age range was from 15-69 years. The mean age of our patients was 35.0 [standard deviation (SD) 13.0]. The mean age for females was 33.0±10.7 and for males 38.1±15.6. 26 (39.4%) patients were under the age 29 years, 27 (40.9%) patients were between 30-49 years and 13 (19.7%) patients were between 50-70 years (table 2). 

**Table 2:** Prevalence of signs and symptoms of Graves’ ophthalmopathy in different age groups

** Age group**	**<29** **n=26**	**30-49** **n=31**	**50-70** **n=13**
**Variable**			
**Sex**			
male female	10 (38.5) 16 (36.4)	7 (50) 11 (36.7)	16 (36.4)
NO SPECS score (mean±SD)	2.4±0.85	2.39±0.84	3.3±1.43
EOM Limit *	0	0	6
Increased IOP** n (%)	6 (40)	5 (33.3)	4 (26.7)
Dry eye n (%)	2 (16.7)	7 (58.3)	3 (25)

* Extraocular Motility limitation

** Intraocular Pressure

Overall, in this study, there were more females involved with TED than males (F/M=1.7/1). The female to male ratio was 1.6 and 2.25 in the younger and the middle age groups respectively, but was 0.6 in the older age group. Even though in the older age group there were more males than females, 8 (61.5%) to 5 (38.5%), this reversal of ratio in older patients was not significant (P=0.17). 

**Fig 1:**
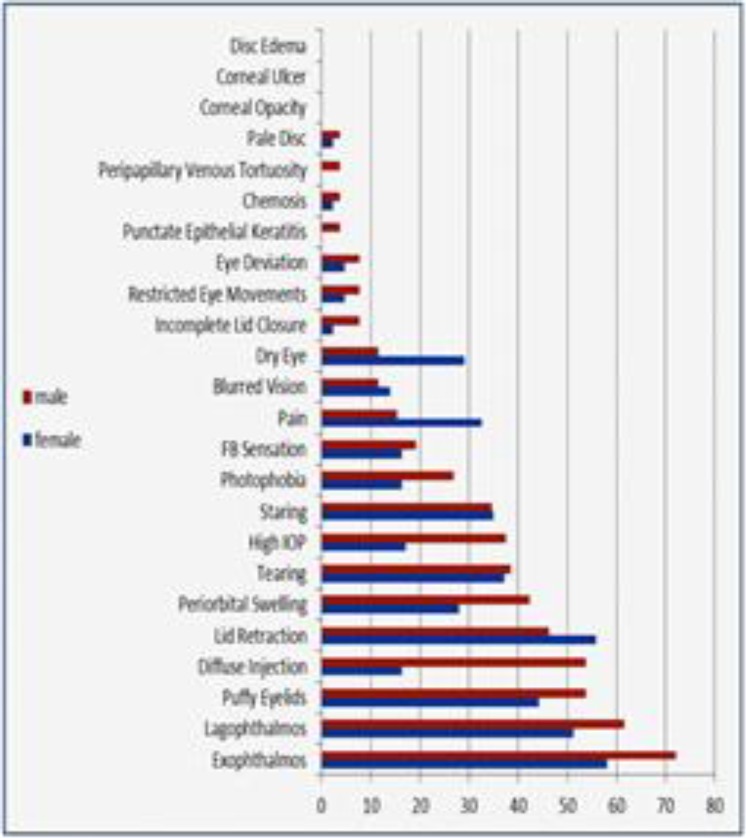
Prevalence of ocular signs and symptoms in 70 patients with Graves’ ophthalmopathy by gender

**Fig 2:**
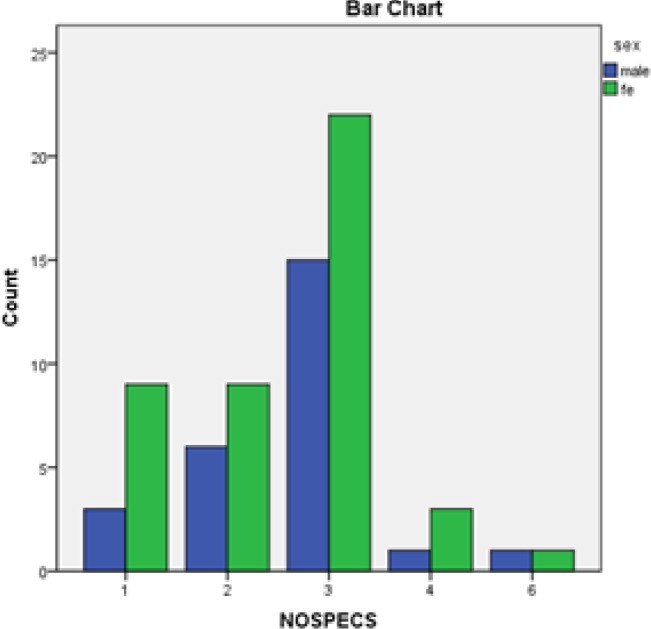
Distribution of 70 patients with Graves’ ophthalmopathy according to NO SPECS score.

The most common ocular finding in this study was proptosis in 44 (63.8%) patients that was unilateral in 14 (31.8%) and bilateral in 30 (68.2%). The next common finding was lid lag in 39 (55.7%) patients, of which 10 (14.3%) were unilateral and 29 (41.4%) were bilateral, and lid retraction in 37 (52.8%) patients of which 29 (41.4%) were bilateral. Other signs and symptoms include tearing in 27 (38.6%), eyelid swelling in 23 (32.9%), conjunctival injection 21 (30%), ocular pain in 18 (25.7%) and dry eye in 12 (17.1%). Not all findings were similar in men and women: eyelid swelling (42.3%) and conjunctival injection (53.8%) was more frequent in males whereas ocular pain (32.6%) and dry eye (20.5%) were seen more in females (fig 1). Ophthalmopathy was classified according to modified Werner’s NO SPECS classification score (table 1). The majority of patients (n=37, 52.9%) had a score of 3, followed by score of 2 (n=15, 21.4%) and score of 1 (n=12, 17.1%) and only 6 (8.6%) patients were 4 or higher (fig 2). There was no significant difference in the scores according to sex (P=0.57) or increasing age (P=0.068).

The mean NO SPECS score for males was 2.69±1.01, and for females 2.52±1.04 but the difference was not found to be significant (P=0.5). The average number of ocular signs and symptoms per patient was 4.1 in those younger than 29 years, 4.0 in those between 30-49 years, and 6.7 in those above 50 years (table 2). This difference was clinically significant (P= 0.004).

The average number of signs and symptoms was greater in males (5.15) than females (4.27), (P=0.17). Clinical severity, as judged by the increasing number of signs and symptoms, was correlated to increasing age (r=0.31, P=0.01). Elevated IOP was seen in 15 (25.4%) patients, 9 (37.5%) males and 6 (17%) females, however the sex difference was not found to be significant (P=0.07). There were 13 (39.4%) patients with proptosis and increased IOP. There was a positive correlation between increased IOP and exophthalmos (P=0.007) chi squared test. Also, there was a positive correlation between increased IOP and severity of disease. In patients with increased IOP, the mean NO SPECS score was 3.13 and the median was 3. In patients with normal IOP mean NO SPECS was 2.3 and the median was 2. This difference was significant (P=0.029).

Patients with elevated IOP had a greater number of signs and symptoms (4.9±2.1) compared to those with normal IOP (4.8±2.7) but the difference was not significant (P= 0.9) (table 2). Limitation of extraocular motility and deviation of the eyes were seen in 6 of our patients, 3 (6.8%) females and 3 males (11.5%). All 6 patients were above 50 years of age. Clinical evidence of dry eye was seen in 12 (17.1%) patients, 3 (25%) males and 9 (75%) females. Foreign body sensation was present in 13 (18.6%) of patients. There was a positive correlation between symptoms of foreign body sensation and clinical evidence of dry eye (P=0.039). However proptosis was not related to dry eye (P=0.74) or foreign body sensation (P=0.34).

In 65% of patients, the presence of TED was concurrent or within 6 months of diagnosis of thyroid disease, 20% between 7-24 months and 15% more than 24 months. There was no correlation between duration of disease and proptosis (P=0.47) or clinical severity (P=0.84). We had only two smokers in this study who were both males. In this study, there was only one patient with unilateral pallor of the optic disc due to compressive optic neuropathy and one case of bilateral venous congestion of the optic nerve head. Both patients were males and more than 50 years old. We had no cases of sight loss due to corneal opacification.

## Discussion

Involvement of the eyes in thyroid disease is a well-known entity. In this study, our aim was to find the relative frequencies of various presenting signs and symptoms of TED in a patient population in northern Iran based on different age groups and gender. Most studies around the world have shown a higher female to male ratio (3, 8). In our study, we had a female to male ratio of 1.7 to 1. Besharati (5), Etezad-Razavi (9) and Perros (10) also showed a higher frequency in females but Kashkouli et al. (11) reported more prevalent thyroid eye disease in males. 

More than 80% of our patients were under the age 50. Similarly in a study by Besharati (5), 85.3% of patients were below 50 years old. In most studies, the mean age of involvement with TED was lower in females than males. Etezad-Razavi reported a mean of 34.7 years for females and 44.7 years for males, which is slightly different from our mean of 33.0 and 38.1 years for females and males, respectively. Lee (12) found a mean of 43.9 years in males and 44.4 years in females, while Perros (10) reported an overall higher mean age of involvement in his patients 49.2±13.4 years. The more severe forms of TED were seen in our male population, even though our results were not clinically significant (P=0.06). Jafari et al. (13), Lee et al. (12) and Perros (10) reported a similar finding of increasing severity in male population. 

The majority of our patients had NO SPECS score or 3 or less which was similar to other studies (3, 9). There was an increasing number of individual signs and symptoms with increasing age, showing that older patients tend to develop more severe forms of TED (p=0.004). The studies by Besharati (5), Perros (10) and Lee (12) also demonstrated this trend. It appears that even though TED is more frequently seen in females, a more aggressive form is seen with increasing age and the male gender (14).

Proptosis was the most common finding in our series (63.8%), followed by lid lag (55.7%), and lid retraction (52.8%). Etezad-Razavi (9) reported lid retraction (64.2%), proptosis (53%) and lid lag (28.1%). Besharati (5) showed proptosis in 52%, lid retraction in 48.5%, and lid lag in 42.2%. Limitation of ocular motility and eye deviation was seen in 8.6% of our patients. Studies in other parts of Iran have reported restricted motility ranging from 13.3% to 41.3% (9, 11, 13). This wide range of clinical manifestation may represent ethnic or regional differences in the patient population. Also the earlier presentation of patients leading to earlier diagnosis and treatment could be a cause of this variability. In a study by Lim et al., it was shown that proptosis and muscle involvement was less pronounced in Asian patients as compared to other ethnic groups (15). 

One notable finding in this study was elevation of IOP. The orbit is a bony structure and any increase in intraorbital volume will cause both displacement of the globe anteriorly and increased intraocular pressure (16). The association of thyroid eye disease with increased IOP has been known for more than a century (17). Increased IOP in up gaze is commonly seen in TED, however it is not specific to TED, and can be seen in any infiltrative orbitopathy due to mechanical compression of the globe (16) In addition, it has been proposed that thyroid related ocular hypertension may be caused by increased episcleral venous pressure or glycosaminoglycan deposition in the trabecular meshwork (16). 

In a study by Haefliger et al. in 1997 (18), performed on 500 patient charts, the prevalence of increased IOP was noted to be 24%. In 2007, Behrouzi et al. (19) reported a prevalence of 11%, and He et al. reported an incidence of 31.3% in Chinese patients (20). With persistence of exophthalmos, there was an increased chance of development and progression of glaucoma signs such as cupping of the disc and visual field defects (20).

In our study, we had 15 (25.4%) cases of increased IOP in primary position. Increase in IOP can be explained by increased pressure on the globe by enlarging muscle masses and by impediment of episcleral outflow. Indeed in our study, there was a strong correlation between proptosis and increase IOP (P=0.007). Increased IOP was seen more in men (37.5%) than women (17.1%), P=0.078. Persistence of TED may lead to progressive increase of IOP, leading to overt manifestations of glaucoma such as cupping of the disc and visual field loss (18), therefore, follow-up of patients in this regard is warranted.

Dry eye has historically been explained by increased tear evaporation from proptotic eyes (21). Nonetheless recently it has been shown that the lacrimal gland may also be a target of TSH antibodies (22). Clinical evidence of dry eye was noted in 12 (17.1%) of our patients. When dry eye was cross-referenced with presence of proptosis, it was not found to be significant (P=0.74), indicating that lacrimal gland dysfunction should be considered as an entity by itself and not merely a consequence of proptosis. Interestingly, it has been shown by Gupta et al. that in patients presenting with dry eyes, previously undiagnosed thyroid dysfunction may be the underlying cause (23).

In our study, we had one case of compressive optic neuropathy that presented with pale discs (2.8%). This patient had severe proptosis, limitation of ocular motility, increased IOP, and periorbital swelling. Kashkouli et al. (11) reported optic nerve involvement in 6.3% of their patients. It has been shown that compressive optic neuropathy is highly correlated to extraocular muscle fibrosis and limitation of movement (24). We did not have enough cases to evaluate this fact independently.

Smoking has been shown to correlate with the severity of TED (25). In our patient population, there were only 2 smokers both of who had severe TED, yet there was not enough data to make a statistical statement in this study. In conclusion, we found that ocular manifestations of Graves' are quite common. Proptosis was the most common ocular finding in this study followed by lid lag and lid retraction. More than 80% of our patients were below age 50, but more severe forms of the disease were seen in older age groups. Dry eye and increased IOP are commonly seen outcomes that should be managed diligently. This potentially sight-threatening condition is seen worldwide and has many functional and cosmetic consequences that need to be recognized.
